# Molecular Imaging of Invasive Pulmonary Aspergillosis Using ImmunoPET/MRI: The Future Looks Bright

**DOI:** 10.3389/fmicb.2018.00691

**Published:** 2018-04-09

**Authors:** Christopher R. Thornton

**Affiliations:** ^1^Department of Biosciences, College of Life and Environmental Sciences, University of Exeter, Exeter, United Kingdom; ^2^ISCA Diagnostics Ltd., Exeter, United Kingdom

**Keywords:** monoclonal antibody (mAb), invasive pulmonary aspergillosis, positron emission tomography, magnetic resonance imaging, immunoPET-MRI

## Abstract

Invasive pulmonary aspergillosis (IPA) is a life-threatening lung disease of immuno-compromised humans caused by the ubiquitous environmental mold *Aspergillus*. Biomarker tests for the disease lack sensitivity and specificity, and culture of the fungus from invasive lung biopsy is slow, insensitive, and undesirable in critically ill patients. A computed tomogram (CT) of the chest offers a simple non-intrusive diagnostic procedure for rapid decision making, and so is used in many hematology units to drive antifungal treatment. However, radiological indicators that raise the suspicion of IPA are either transient signs in the early stages of the disease or not specific for *Aspergillus* infection, with other angio-invasive molds or bacterial pathogens producing comparable radiological manifestations in a chest CT. Improvements to the specificity of radiographic imaging of IPA have been attempted by coupling CT and positron emission tomography (PET) with [^18^F]fluorodeoxyglucose ([^18^F]FDG), a marker of metabolic activity well suited to cancer imaging, but with limited use in invasive fungal disease diagnostics due to its inability to differentiate between infectious etiologies, cancer, and inflammation. Bioluminescence imaging using single genetically modified strains of *Aspergillus fumigatus* has enabled *in vivo* monitoring of IPA in animal models of disease. For *in vivo* detection of *Aspergillus* lung infections in humans, radiolabeled *Aspergillus*-specific monoclonal antibodies, and iron siderophores, hold enormous potential for clinical diagnosis. This review examines the different experimental technologies used to image IPA, and recent advances in state-of-the-art molecular imaging of IPA using antibody-guided PET/magnetic resonance imaging (immunoPET/MRI).

## Introduction

Invasive pulmonary aspergillosis is a frequently fatal lung disease of immuno-compromised individuals caused by inhalation of spores of the air-borne fungus *Aspergillus*. While a number of species of *Aspergillus* can cause IPA, the principal agent of the disease is *Aspergillus fumigatus*, responsible for >80% of recorded cases. As an opportunistic pathogen, it causes more than 200,000 life-threatening infections in humans every year, mainly in high-risk patient groups such as those with hematological malignancies, and in hematopoietic stem cell (HSC) and solid organ transplant recipients, with mortality rates of between 30 and 90% (Segal, 2009). The disease is typically seen during periods of prolonged neutropenia, but non-neutropenic patients with underlying lung diseases such as steroid-treated chronic obstructive pulmonary disease (COPD), asthma, lung cancer, or autoimmune diseases with pulmonary involvement can develop IPA (Prattes et al., 2014). Furthermore, other *Aspergillus* lung diseases such as chronic pulmonary aspergillosis (CPA) and allergic bronchopulmonary aspergillosis (ABPA) are thought to affect >5 million people globally each year (Brown et al., 2012). Taken together, *Aspergillus* diseases represent a significant burden to human health, contributing to patient morbidities and prolonging hospitalization. Much of this burden is caused by the lack of diagnostic tests with sufficient accuracy to allow early identification and timely intervention with effective antifungal drugs.

Early detection of IPA and treatment with mold-active drugs is vital for patient survival. However, clinical symptoms of the disease (fevers and chills, hemoptysis, shortness of breath, chest pains, and headaches) are not specific for *Aspergillus* infections. The gold standard test for IPA is culture of *Aspergillus* from a sterile biopsy, but this is limited by poor sensitivity, lengthy turnaround time, and requires invasive recovery of lung tissue. Assays that detect circulating biomarkers of infection such as the Platelia galactomannan enzyme-linked immunosorbent assay (ELISA) and “pan-fungal” β-D-glucan tests lack either sensitivity or specificity (Prattes et al., 2014). The *Aspergillus* lateral-flow assay (LFA; Thornton, 2008) will be available commercially as a CE-marked *in vitro* device (IVD) for IPA diagnosis in March 2018. When used with BAL samples, it has the ability to be used as a point-of-care test, and so has the potential to improve the speed and accuracy of disease detection (Hoenigl et al., 2017). Despite this, the current inadequacies of IPA diagnostics have led to the empiric or “fever-driven” use of antifungals. This contributes to the erroneous treatment of already sick patients with costly and noxious drugs and promotes the emergence in *Aspergillus* of resistance to mold-active triazoles and to breakthrough infections. Empiric antifungal treatments also impact the sensitivities of fungal culture and biomarker-assisted tests, which are needed for diagnosis, for establishing drug sensitivities, and for monitoring responsiveness to treatments.

Diagnostic-driven approaches to antifungal treatment have been shown to be more effective than empiric treatment with respect to both cost and patient outcome (Barnes, 2013). Diagnostic-driven approaches to IPA treatment habitually rely on radiographic imaging, coupled with frequent testing for fungal biomarkers. Radiographic imaging is an attractive means of detecting *Aspergillus* lung infections *in vivo* because it is a non-invasive procedure, but basic radiographic findings in IPA are largely non-specific (Panse et al., 2016). A computed tomogram (CT) of the chest provides a quick non-intrusive clue for rapid decision making (Prasad et al., 2016), with the earliest sign suggestive of the disease being a nodule. The “halo sign,” a transient CT finding, is also suggestive of probable disease, and initiation of antifungal treatment in patients with this indicator at baseline has been associated with improved patient outcomes for early stages compared to later stages of disease (Greene, 2005; Greene et al., 2007). However, other mold pathogens such as mucormycetes species, and angio-invasive bacterial pathogens such as *Pseudomonas aeruginosa*, can give similar appearances (Segal, 2009; Stanzani et al., 2015). The “halo sign” therefore provides only limited accuracy for the diagnosis of IPA.

Over recent years, alternative techniques for non-invasive imaging of IPA have been developed and tested in pre-clinical animal models of disease (Velde and Wiehr, 2017). One such method is bioluminescence, that has been used to track *Candida albicans* and *A. fumigatus* infections and to monitor their responsiveness to antifungal treatments (Doyle et al., 2006; d’Enfert et al., 2010; Brock, 2012; Jacobsen et al., 2014). Bioluminescent strains of *A. fumigatus* have been generated through constitutive expression of the firefly luciferase gene under the fungal promoter *gpdA* (Brock et al., 2008). Transformed strains of the pathogen have been used to monitor antifungal drug efficacies *in vitro* and *in vivo* (Brock et al., 2008; Galiger et al., 2013) and to investigate the roles of resident and recruited immune effector cells in defense against invasive *A. fumigatus* infections (Ibrahim-Granet et al., 2010). The limitation of this technique is the requirement for genetically modified strains, which restricts studies to single mutants of the pathogen expressing luciferase. Different approaches for imaging IPA have therefore been explored using, for example, small molecules such as peptides (Yang et al., 2009), and the antifungal drug fluconazole coupled to ^18^F or ^99m^Tc (Lupetti et al., 2002), for scintigraphic imaging of infections. For instance, using a ^111^In-labeled peptide c(CGGRLGPFC)-NH_2_ selected from a bacteriophage phage library, γ-imaging is able to delineate experimental IPA in mice (Yang et al., 2009). However, because the peptide corresponds to extracellular matrix proteins of the lung parenchyma, it is probable that the peptide binds to other fungi that are able to interact with extracellular matrix components of the lungs. Further specificity tests would therefore need to be conducted *in vivo* to determine the spectrum of IFDs detectable with this probe. While ^99m^Tc-fluconazole proved to be superior to ^18^F-fluconazole for imaging of *C. albicans* infections in mice, it was found to be unsuitable for imaging of *A. fumigatus* infections (Lupetti et al., 2002).

The limitations of bioluminescence and small molecule imaging have led to efforts to improve the specificity of radiographic imaging of IPA by combining well-established hospital imaging technologies [high-resolution computed tomography (HRCT), MRI, and PET] with specific markers of infection. The aim of this mini-review is to examine recent advances in molecular imaging of IPA using radiolabeled *Aspergillus*-specific monoclonal antibodies (mAbs), and iron siderophores, and their potential for translation to the clinical setting.

## Aspergillosis Imaging With Computed Tomography and Magnetic Resonance Imaging

According to current EORTC/MSG guidelines (De Pauw et al., 2008), a CT examination of the chest that reveals “dense, well-delineated, nodular infiltrates in the lung with or without ground glass attenuation (‘halo sign’) in a patient with an ongoing or recent history of prolonged neutropenia, or hematopoietic stem cell transplant (HSCT),” is defined as having a “possible” mold infection. Importantly, while 88–96% of neutropenic patients exhibit this sign in the first day of IPA, it disappears in one-third of patients within 72 h and in the remaining two-thirds of patients within 2 weeks (Caillot et al., 1997; Brodoefel et al., 2006). Furthermore, this diagnosis is not specific for IPA, as other infections, and neoplastic and inflammatory processes, can produce similar opacities with ground glass attenuation (Georgiadou et al., 2011), and the disease can also manifest as atypical presentations in liver transplant patients (Mucha et al., 2013) and during invasive bronchial-pulmonary aspergillosis in critically ill patients with COPD (Huang et al., 2016). Radiological indications are rare in the initial stages of IPA in non-neutropenic patients (Prattes et al., 2014), and differ between children and adults with IFDs (Ankrah et al., 2016).

Notwithstanding these limitations, CT acts as a prompt for instigating antifungal treatment in numerous hematology units, with HRCT providing opportunities for improved antifungal stewardship (Stanzani et al., 2016). Reductions in the unnecessary use of antifungal drugs have been reported in centers using HRCT to drive treatment strategies in allogeneic transplant patients who have persistent febrile antibiotic-resistant neutropenia (Dignan et al., 2009). Therefore, in certain settings, refractory fever/HRCT-based approaches to diagnosis may result in significant reductions in parenteral antifungal drug usage in patients who would otherwise have received empirical treatment. However, due to the limited specificity of HRCT, such an approach cannot be used for IPA specifically, but rather IFDs as a whole (Dignan et al., 2009). Improvements to the specificity of HRCT for the detection of fungal lung infections have been attempted by combining it with pulmonary angiography (CTPA), but the performance of CTPA relative to other signs (halo sign, hypo-dense sign, pleural effusion, and reversed halo sign) is not known. Nevertheless, vessel occlusion detected by CTPA may be a more sensitive and possibly more specific radiographic sign in patients with hematological malignancies (Stanzani et al., 2015).

Pre-clinical studies using MORF oligomers that target fungal ribosomal RNA have shown that CT specificity can be dramatically improved when ^99m^Tc-labeled *Aspergillus*-specific probes are combined with SPECT (Wang et al., 2013). Two probes, AGEN and AFUM, have been investigated that are genus-specific and *A. fumigatus*-specific, respectively. Single photon emission tomography (SPECT)/CT imaging of mice with experimental IPA showed a twofold increased accumulation of both ^99m^Tc-labeled probes in *A. fumigatus* infected lungs compared to uninfected controls. While the AGEN oligomer was found to cross-react with *C. albicans*, and the AFUM oligomer would preclude detection of infectious *Aspergillus* species other than *A. fumigatus*, the work nevertheless elegantly demonstrates that CT imaging can be rendered disease-specific by using pathogen-specific probes.

Magnetic resonance imaging is now the non-invasive imaging tool of choice, with high spatial resolution and the highest soft tissue contrast. However, unlike HRCT, MRI has received far less attention as a diagnostic imaging modality for *Aspergillus* lung infections, but has been studied extensively as a detection aid for *Aspergillus* cerebral and central nervous system infections (Starkey et al., 2014; Marzolf et al., 2016). While CT is highly suitable for lung applications because it produces high-resolution 3D images with an excellent air–tissue contrast, MRI of the lung is challenging owing to the lack of detectable protons in air-filled spaces and potential artifacts between air–tissue interfaces. Despite these shortcomings, a longitudinal *in vivo* study in mice showed that *A. fumigatus* lung lesions could be visualized and quantified using MRI (Poelmans et al., 2016). By using an advanced MR pulse sequence with ultra-short echo times, pathological changes within the infected lung were visually and quantitatively detectable and with a high degree of sensitivity. In humans, dynamic contrast-enhanced MRI (DCE-MRI) might also be useful for imaging IPA in immunosuppressed acute myeloid leukemia patients (Araz et al., 2014).

## Aspergillosis Imaging With [^18^F]FDG Positron Emission Tomography

In contrast to HRCT and MRI, PET can give information about the physiological status of the particular target organ. It has emerged as an immensely powerful imaging technique in the field of oncology, but its use in infectious disease imaging is very much in its infancy (Glaudemans et al., 2012, 2015; Signore et al., 2015). Compared to SPECT of IPA using ^67^Ga scintigraphy (Tzen et al., 1999; Goméz et al., 2000), PET provides increased sensitivity and resolution through coincidence detection of photons emitted from radionuclei resulting from positron annihilation, with its success in oncology resulting from the use of [^18^F]fluorodeoxyglucose ([^18^F]FDG), a diagnostic tracer that specifically accumulates in metabolically active inflammatory cells (neutrophils, macrophages, and lymphocytes), cancer cells, and during infectious processes (Imperiale et al., 2010; Bassetti et al., 2017).

Several studies have indicated that [^18^F]FDG might be useful for imaging fungal infections (Sharma et al., 2014), for differentiating between non-invasive and invasive aspergillosis (Kim et al., 2013), for identification of extra-pulmonary sites of infection (Chamilos et al., 2008), and for therapy monitoring (Ho et al., 1998; Ozsahin et al., 1998; Franzius et al., 2001; Stanzani et al., 2016). However, a recent study by Rolle et al. (2016), which employed PET and MRI (PET/MRI) to detect lung infections by *A. fumigatus in vivo*, showed that the increase in [^18^F]FDG uptake during *Aspergillus* lung infection could not be distinguished from that seen during sterile inflammation or during bacterial lung infections caused by *Streptococcus pneumonia* or *Yersinia enterocolitica*, further demonstrating the lack of specificity of this tracer which has been reported elsewhere (Petrik et al., 2014). While these studies are pre-clinical investigations using mouse models of IPA, numerous clinical studies have also reported the lack of specificity of FDG-PET for diagnosing the disease in humans, with *Aspergillus* lung diseases mimicking lung cancer (Wilkinson et al., 2003; Baxter et al., 2011; Garcia-Olivé et al., 2011), metastatic thyroid cancer (Ahn et al., 2011), lymphoma (Sonet et al., 2007), and tuberculoma (Nishikawa et al., 2011). Pulmonary IFIs caused by fungi other than *Aspergillus* (e.g., *Candida*, *Blastomyces*, *Cryptococcus*, *Coccidioides*, *Histoplasma*, mucormycetes, and *Pneumocystis*) are also detected by FDG-PET in humans (Ankrah et al., 2016). Consequently, while FDG-PET may confirm IPA lesions observed using HRCT and other imaging modalities (Chamilos et al., 2008; Hot et al., 2011), and to guide needle aspirations of lung tissues for fungal culture (Casal et al., 2009), it cannot be used for definitive *in vivo* diagnosis of IPA, or for its differentiation from ABPA (Nakajima et al., 2009) or aspergillomas (Franzius et al., 2001; Ahn et al., 2011).

## Aspergillosis Imaging With ^68^Ga-Siderophores

Other than the *Aspergillus*-reactive ^99m^Tc-labeled MORF probes (Wang et al., 2013), few imaging tracers have been developed that specifically target *Aspergillus* infections, but substantial success has been achieved by combining microPET/CT with iron-scavenging siderophores labeled with ^68^Ga or ^89^Zr. Iron is essential for fungal growth and, in iron-poor environments such as serum, bacteria and fungi produce low molecular weight ferric iron-specific chelators to scavenge iron from the host (Haas, 2003). *A. fumigatus* lacks specific uptake systems for host iron sequestered in heme, ferritin or transferrin, and instead uses two high-affinity iron uptake mechanisms, reductive iron assimilation and siderophore-assisted iron mobilization, both of which are induced under conditions of iron starvation. The pathogen produces three hydroxamate-type siderophores, extracellular fusarinine C (FSC) and triacetylfusarinine C (TAFC), and intracellular ferricrocin (FC; Schrettl et al., 2004; Haas et al., 2015). TAFC is secreted soon after spore germination in iron-limited media (Hissen et al., 2004), and is detectable in serum from patients with proven/probable IPA (Carroll et al., 2016). Its biosynthesis is an essential requirement for spore germination, and for virulence in a mouse model of disease (Schrettl et al., 2004; Hissen et al., 2005). Using ^68^Ga, a positron emitter with complexing properties similar to those of Fe(III), Petrik et al. (2010) evaluated the potential of [^68^Ga]TAFC and [^68^Ga]FC as radiopharmaceuticals for imaging of IPA. They showed that uptake by *A. fumigatus* was highly dependent on iron load, but that [^68^Ga]TAFC displayed excellent *in vitro* stability, and highly selective accumulation in iron-starved cells. Uptake of [^68^Ga]TAFC in the lungs of immunosuppressed rats correlated with severity of *Aspergillus* infection, while the bio-distribution of [^68^Ga]FC was inferior to [^68^Ga]TAFC, and showed poor stability both *in vitro* and *in vivo*. [^68^Ga]TAFC was again shown in a subsequent pre-clinical study to selectively accumulate in infected lung tissues in a rat infection model, and that another siderophore ferrioxamine (FOXE), when coupled to ^68^Ga ([^68^Ga]FOXE), also displayed excellent pharmacokinetics, highly selective accumulation in *Aspergillus* infected lung tissues, and similarly good correlation with disease severity (Petrik et al., 2012a,b). A downside of using the radionuclide ^68^Ga in PET imaging, compared to longer-lived positron emitters such as ^64^Cu (*t*1/2 = 12.7 h), ^124^I, ^86^Y, ^90^Nb, or ^89^Zr (*t*1/2 = 78.41 h), is its relatively short half-life (*t*1/2 = 67.7 min), which limits its use in longitudinal studies. For this reason, alternative siderophores have been investigated for radiolabeling with ^89^Zr and for use as imaging agent for *Aspergillus* infections (Petrik et al., 2016). Small animal imaging studies of all ^68^Ga- and ^89^Zr-labeled siderophores injected in mice displayed similar pharmacokinetics and minimal accumulation of radioactivity in blood and other organs and tissues, with the exception of [^89^Zr]FOXE which caused significant retention in the gastrointestinal tract. [^89^Zr]TAFC showed favorable properties for potential longitudinal *Aspergillus* infection imaging.

Using the radiolabeled siderophores as diagnostic tracers should allow for highly specific detection of IPA since TAFC and FC have no function in human physiology and uptake is not detectable in human lung cancer cells (Petrik et al., 2014). However, while the energy-dependent siderophore transporter system might appear advantageous compared to cell wall-labeling approaches using, for example, *Aspergillus*-specific mAbs, the use of radiolabeled siderophores does have its limitations. The first is specificity. While pathogenic bacteria have been shown not to use TAFC- or FOXE-mediated uptake of iron (Petrik et al., 2014), Fe-TAFC uptake under iron depletion has been demonstrated in the human pathogenic fungi *C. albicans* (Lesuisse et al., 2002) and *Fusarium oxysporum* (Leal et al., 2013), and [^68^Ga]TAFC and [^68^Ga]FOXE uptake under iron deficiency has been shown in the human pathogens *Fusarium solani* and *Rhizopus oryzae* (Petrik et al., 2014). Consequently, further work is needed to determine whether [^68^Ga]TAFC (or [^68^Ga]FOXE) uptake is able to discriminate between *Aspergillus* infections *in vivo* and commensal *C. albicans* colonization of the gastrointestinal tract, invasive candidiasis, mucormycosis, and disseminated *Fusarium* infections (fusariosis). Furthermore, while *A. fumigatus* is the principal agent of IPA, other *Aspergillus* species such as *Aspergillus flavus*, *Aspergillus nidulans*, *Aspergillus niger*, and *Aspergillus terreus* are able to cause the disease (Willinger et al., 2014). While uptake of Fe-TAFC has been shown in *A. nidulans* (Oberegger et al., 2001) and [^68^Ga]TAFC uptake has been shown in *A. flavus* and *A. terreus* (albeit at significantly lower levels than *A. fumigatus*), it is unclear whether all infectious *Aspergillus* species are detectable *in vivo* using this system. The second consideration is the important role of iron overload in the development of invasive fungal diseases. Many patients at high risk for developing IPA (heavily transfused AML patients, neutropenic patients, liver and allogeneic HSCT recipients, and those receiving chemotherapy) frequently have iron overload, which has been shown to contribute to the increased susceptibility of these groups to invasive fungal infections (Alexander et al., 2006; Bullen et al., 2006; Pagano et al., 2011). In these patients, freely available iron could arguably lead to decreased imaging sensitivity using [^68^Ga]TAFC (or [^68^Ga]FOXE), given the strong correlation between iron availability and uptake of radiolabeled siderophores by *A. fumigatus*. Despite these potential drawbacks, the possibility of accurately diagnosing IPA in humans using [^68^Ga]TAFC or [^68^Ga]FOXE imaging merits clinical evaluation.

## Aspergillosis Imaging With Monoclonal Antibody JF5

Despite the abilities of mAbs to differentiate different genera and species of human pathogenic fungi, and their capacity to discriminate between inactive spores and invasive hyphae (Thornton and Wills, 2015), their use in molecular imaging of IFDs has yet to be fully realized. Imaging with antibodies has, until very recently, been the domain of cancer detection (Mestel, 2017), with limited application in infectious disease diagnostics (Rolle and Wiehr, 2017). However, recent studies have demonstrated the enormous potential of antibody-guided PET/MRI (immunoPET/MRI) technologies to dramatically improve the molecular imaging of viral (Santangelo et al., 2014), bacterial (Wiehr et al., 2016), and fungal (Rolle et al., 2016; Davies et al., 2017) infections *in vivo*, with the real possibility of precision medicine for infectious diseases in the near future (Jain, 2017). ImmunoPET/MRI marries functionality of PET with the specificity of mAbs and anatomical depiction of MRI. Any infectious disease can potentially be detected with this technology provided that high-integrity antibodies are available that have sufficient specificity for the target organism and which detect signature molecules of active infection. In the case of IPA, these diagnostic requirements have been met through the *Aspergillus*-specific mouse mAb mJF5 (Thornton, 2008) and its humanized derivative hJF5 (Davies et al., 2017). mAb mJF5, which forms the basis of the *Aspergillus* LFA (Thornton, 2008; Prattes et al., 2014; Hoenigl et al., 2017), binds to extracellular (galacto)mannoprotein antigens produced by all clinically relevant *Aspergillus* species (Thornton, 2014; Davies et al., 2017), and is able to detect IPA in humans caused by *A. fumigatus*, *A. flavus*, *A. nidulans*, *A. niger*, and *A. terreus*, either as single or as mixed species infections (Willinger et al., 2014). Its high-level specificity for *Aspergillus* species means that it is able to discriminate between IPA and lungs infections caused by other mold pathogens including *F. solani* (Willinger et al., 2014). Furthermore, the JF5 antigen is produced during active growth only, which means that it is able to differentiate between inactive spores present in inhaled air and invasive hyphae that infect or colonize the lung (Thornton, 2008, 2014).

## Aspergillosis Imaging With Antibody-Guided Positron Emission Tomography/Magnetic Resonance Imaging

Novel probes for the non-invasive detection of *A. fumigatus* lung infection based on antibody-guided PET/MR imaging (immunoPET/MRI) have recently been reported (Rolle et al., 2016; Davies et al., 2017). Administration of [^64^Cu]DOTA-labeled mAb mJF5 to neutrophil-depleted *A. fumigatus*-infected mice allowed specific localization of lung infections when combined with PET, while optical imaging with a fluorophore (DyLight650)-labeled version of the mAb showed co-localization with invasive hyphae (Rolle et al., 2016). The [^64^Cu]DOTA-mJF5 tracer distinguished *Aspergillus* from bacterial lung infections and, unlike [^18^F]FDG-PET, differentiated *Aspergillus* infection from lung inflammation caused by a sterile inflammatory stimulus. The long *in vivo* half-life of [^64^Cu]DOTA-mJF5 allows repeat imaging of infections following a single injection of the radioactive tracer and, because it is hyphal-specific, may prove useful in monitoring infection in response to antifungal treatment.

Despite specific uptake of the [^64^Cu]DOTA-mJF5 tracer in the lungs of *A. fumigatus*-infected animals compared to uninfected controls, high liver uptake of the tracer was also evident. It was hypothesized that this liver uptake might be due to hepatic removal of the radiolabeled antibody circulating in the bloodstream, specific binding to antigen in liver tissues following shedding of soluble antigen from hyphae in the lungs, and transchelation of ^64^Cu to liver proteins due to insufficiently strong binding to the chelator DOTA. Transchelation of ^64^Cu to serum protein was shown not to occur, although it has been shown elsewhere that DOTA has a poor *in vivo* stability, which results in loss of the radio-metal and its non-specific accumulation in off-target tissues. In a subsequent study (Davies et al., 2017), the chelator DOTA was exchanged with the alternative ^64^Cu chelators DOTAGA and NODAGA, which have increased *in vivo* stability. This decreased the uptake of the radiolabeled immuno-conjugates in the liver, while preserving specific accumulation in the *A. fumigatus*-infected lung. In particular, a NODAGA conjugated tracer ([^64^Cu]NODAGA-mJF5) provided the lowest liver uptake, while enabling the greatest uptake in infected lungs.

**FIGURE 1:**
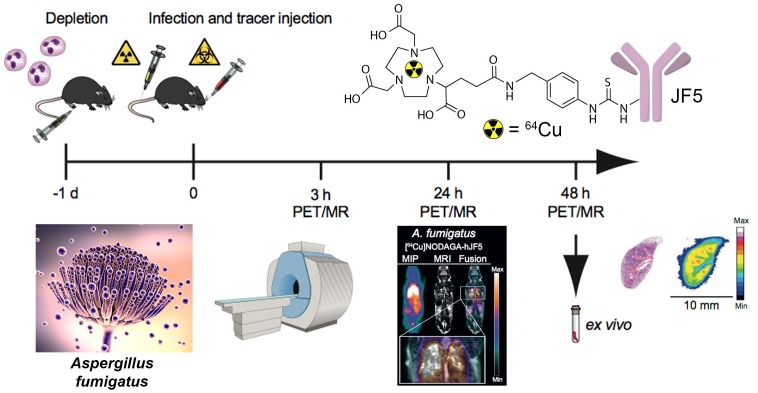
Schematic representation of antibody-guided immunoPET/MR imaging of IPA in a neutropenic mouse model of disease. Twenty-four hours (–1 day) prior to infection with *A. fumigatus*, and administration of the PET tracer, neutropenia is induced in mice by the injection of 100 μg of the anti-Ly-6G/anti-Ly6C antibody RB6-8C5. At time 0, mice are infected by intratracheal injection with an *A. fumigatus* spore suspension and with simultaneous tail vein injection with a JF5-based PET tracer. The tracer shown is mAb JF5 conjugated to the positron emitter ^64^Cu using the chelator NODAGA. Simultaneous PET/MR imaging of animals is then performed 3, 24, and 48 h after infection/tracer injection. *Ex vivo* bio-distribution and autoradiography are conducted after the last PET scan at 48 h. Adapted from Davies et al. (2017).

**FIGURE 2:**
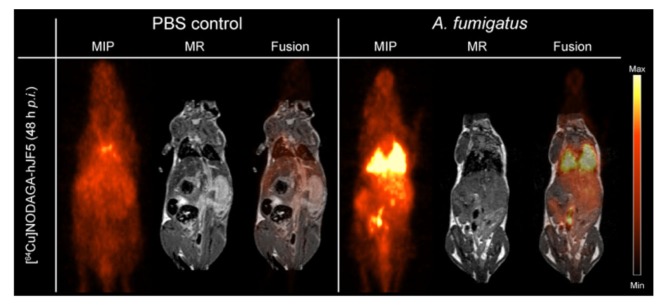
ImmunoPET/MR imaging of IPA using the humanized *Aspergillus*-specific tracer [^64^Cu]NODAGA-hJF5. The images show coronal maximum intensity projections (MIP), magnetic resonance (MR) imaging, and fused positron emission tomography (PET)/MR images of uninfected mice (PBS control) and mice infected with *A. fumigatus*. In order to render animals neutropenic, they received an intraperitoneal injection of the antibody RB6-8C5 and, 24 h later, were injected with the pathogen and [^64^Cu]NODAGA-hJF5 tracer, according to the experimental procedure shown in **Figure 1**. Forty-eight hours after injection with the pathogen and humanized tracer, PET/MR imaging shows specific accumulation of the tracer in *A. fumigatus*-infected lung tissues. Image courtesy of MATHIAS Consortium.

The immunoPET/MR imaging technology based on mAb mJF5 is fundamentally translatable to human disease detection since the antibody tracks a biomarker of *Aspergillus* infection that has been clinically validated for human IPA diagnosis using LFA tests of serum and BAL fluids (Thornton, 2008; Held et al., 2013; White et al., 2013; Prattes et al., 2014; Willinger et al., 2014; Hoenigl et al., 2017). To allow translation of the antibody tracer to the clinical setting, a humanized version of the antibody (hJF5) has been generated by grafting of the mJF5 CDRs into a human IgG1 framework (Davies et al., 2017). Pre-clinical imaging with a [^64^Cu]NODAGA-hJF5 tracer in a neutropenic mouse model of IPA (**Figure 1**) has demonstrated improved PET/MR image resolution of *A. fumigatus* lung infections compared to its murine counterpart [^64^Cu]NODAGA-mJF5 (Davies et al., 2017), with the lowest liver uptake but highest uptake in infected lungs (**Figure 2**).

Using targeted deletion of the gene encoding UDP-galactopyranose mutase, an enzyme involved in the production of galactofuranose-containing glyco-conjugates in *Aspergillus* species, mAb JF5 has been shown to bind to β1,5-galactofuranose (Gal*f*), an immuno-dominant epitope present in its (galacto)mannoprotein target (Davies et al., 2017). The absence of the epitope Gal*f* in mammalian carbohydrates (Tefsen et al., 2012), in addition to the enhanced imaging capabilities of the hJF5 antibody, reduces the likelihood of the [^64^Cu]NODAGA-hJF5 tracer binding to human structures non-specifically, while providing a highly sensitive, non-invasive, procedure for visualizing real-time *Aspergillus* infections of the human lung.

While the pre-clinical imaging studies in the mouse model of disease have shown the enormous potential of the humanized antibody tracer to detect IPA in the context of neutropenia, a number of issues have yet to be resolved. The ability of the tracer to detect chronic semi-invasive aspergillosis syndromes in the setting of underlying lung diseases such as COPD and cystic fibrosis has yet to be established, as has its ability to detect IPA in immunocompetent patients presenting with diffuse bilateral chest infiltrates, with or without cavity (Pathak et al., 2011). In addition, while pre-clinical studies have demonstrated excellent performance of the JF5 tracer in detecting *Aspergillus* lung infections under high inoculum load, its capability in detecting extra-pulmonary infections involving, for example, the brain, spleen, and bone (Davoudi et al., 2014), and its ability to penetrate necrotic tissues, also needs to be determined. However, given the very low background uptake of the tracer in these organs (Rolle et al., 2016; Davies et al., 2017; **Figure 2**), it is likely that the [^64^Cu]NODAGA-hJF5 tracer will be able to detect deep-seated infections in tissues other than the lung.

Despite these caveats, the humanized JF5 antibody represents an ideal candidate for molecular imaging of IPA in humans and translation of the antibody-guided imaging technology to the clinical setting. To this end, the NODAGA-labeled hJF5 antibody has entered mammalian toxicity testing and will enter first-in-human clinical trials in 2018.

## Conclusion

Clinical diagnosis of IPA remains extremely challenging, with non-specific patient symptoms, and insufficient specificity and sensitivity of diagnostic biomarker tests. Radiological imaging of the lung is an attractive means of diagnosing invasive fungal infections since it is a non-invasive procedure, but abnormalities seen in a chest CT which are suggestive of IPA are not sufficiently specific for definitive diagnosis of the disease. Attempts have been to improve radiological detection of IPA using [^18^F]FDG-PET, but uptake of the tracer during IPA is indistinguishable from that seen during cancer, inflammatory reactions, or during bacterial infections. The specificity of PET has been dramatically improved through the use *Aspergillus* siderophores, and mAbs conjugated to radionuclides, but all studies to date have been conducted in animal models of IPA.

For translation to the clinical setting, the *Aspergillus*-specific mAb JF5 has been humanized using CDR grafting of the mouse IgG3 heavy and light chain variable fragments into a human IgG1 framework. The humanized antibody (hJF5) is currently undergoing toxicity testing prior to clinical trials to allow immunoPET/MR imaging of IPA in humans with the *Aspergillus*-specific PET tracer [^64^Cu]NODAGA-hJF5. Once the accuracy of the tracer has been established in human clinical trials, its cost effectiveness as a hospital diagnostic procedure for IPA, and its usefulness in monitoring disease in humans in response to antifungal treatment, will then need to be established. These are lengthy and costly investigations but, if successful, may herald a new age in molecular imaging of IPA, and could act as a paradigm for antibody-guided imaging of other invasive fungal diseases of humans. As a platform technology, immuno-PET/MR can be used to image any invasive fungal disease providing that well-characterized disease-specific antibodies are available. Highly specific mAbs have been reported for a number of the most serious mold pathogens of humans such as *Fusarium*, *Scedosporium*, and *Lomentospora* (Thornton, 2009, Thornton et al., 2015; Al-Maqtoofi and Thornton, 2016), enabling molecular imaging of invasive diseases (fusariosis and scedosporiosis) caused by these pathogens. However, it is important to note that despite the unparalleled sensitivity and specificity of immuno-PET/MRI, the high cost of its development, clinical evaluation, and implementation in healthcare systems means that it will likely only be accessible in specialist centers, and will not replace but rather complement less sophisticated diagnostic tests such as ELISA, PCR, and LFA.

## Author Contributions

The author confirms being the sole contributor of this work and approved it for publication.

## Conflict of Interest Statement

The author is the Director of the ISCA Diagnostics Ltd. and is the inventor of the patented *Aspergillus*-specific monoclonal antibody JF5 and the *Aspergillus* lateral-flow device.

## Abbreviations

CT: computed tomography
IFD: invasive fungal disease
IPA: invasive pulmonary aspergillosis
MRI: magnetic resonance imaging
PET: positron emission tomography
